# Screening of the Key Genes for the Progression of Liver Cirrhosis to Hepatocellular Carcinoma Based on Bioinformatics

**DOI:** 10.1155/2022/2515513

**Published:** 2022-09-26

**Authors:** Yuanbin Chen, Hongyan Qian, Xiao He, Jing Zhang, Song Xue, Yumeng Wu, Jian Chen, Xuming Wu, Suqing Zhang

**Affiliations:** ^1^Medical School of Nantong University, Jiangsu, Nantong 226000, China; ^2^Key Laboratory of Cancer Research Center Nantong, Affiliated Tumor Hospital of Nantong University, Jiangsu, Nantong 226361, China; ^3^The Immunology Laboratory, Affiliated Tumor Hospital of Nantong University, Jiangsu, Nantong 226361, China; ^4^Nantong Fourth People's Hospital, Jiangsu Nantong 226001, China; ^5^Department of Hepatobiliary and Pancreatic Surgery, Affiliated Tumor Hospital of Nantong University, Jiangsu, Nantong 226361, China

## Abstract

Hepatocellular carcinoma (HCC), which is among the most globally prevalent cancers, is strongly associated with liver cirrhosis. Using a bioinformatics approach, we have identified and investigated the hub genes responsible for the progression of cirrhosis into HCC. We analyzed the Gene Expression Omnibus (GEO) microarray datasets, GSE25097 and GSE17549, to identify differentially expressed genes (DEGs) in these two conditions and also performed protein-protein interaction (PPI) network analysis. STRING database and Cytoscape software were used to analyze the modules and locate hub genes following which the connections between hub genes and the transition from cirrhosis to HCC, progression of HCC, and prognosis of HCC were investigated. We used the Kyoto Encyclopedia of Genes and Genomes (KEGG) analysis to detect the molecular mechanisms underlying the action of the primary hub genes. In all, 239 DEGs were obtained, with 94 of them showing evidence of upregulation and 145 showing evidence of downregulation in HCC tissues as compared to cirrhotic liver tissues. We identified six hub genes, namely, BUB1B, NUSAP1, TTK, HMMR, CCNA2, and KIF2C, which were upregulated and had a high diagnostic value for HCC. Besides, these six hub genes were positively related to immune cell infiltration. Since these genes may play a direct role in the progression of cirrhosis to HCC, they can be considered as potential novel molecular indicators for the onset and development of HCC.

## 1. Introduction

Global rates of morbidity and death caused by hepatocellular carcinoma (HCC), which is among the most common cancers in the world and the second most lethal, are on the rise [1]. Currently, both hepatitis B virus (HBV) and hepatitis C virus (HCV) have been identified as the most important cause of HCC [2, 3]. HCC is most likely to occur in patients with severe HBV infections, especially in those who suffer from posthepatitis cirrhosis. Posthepatitis cirrhosis also raises the incidence rate of hepatic sclerosis to 84.6%, which in turn, raises the incidence of HCC to 49.9% [4].

To successfully prevent, diagnose, and treat HCC, it is crucial to understand how liver cirrhosis transforms into HCC. Many studies have showed that capillarization of liver sinusoidal endothelial cells, portal hypertension, immunosuppressive tumor microenvironment, etc., were important factors promoting the development from liver cirrhosis to HCC [5, 6]. However, mitigating these factors did not change the progression of the disease. And currently, there are no ideal molecular markers that can help in distinguishing HCC from cirrhosis. To close this gap, the molecular aspects of HCC incidence, progress, and reasons for poor prognosis need to be further understood.

In this study, we analyzed two mRNA microarrays from the GEO database to identify differentially expressed genes (DEGs) that vary in expression levels between HCC tissues and cirrhotic liver tissues. Following this, protein-protein interaction (PPI) network analyses and Kaplan-Meier curves investigated the connections between the identified genes and those between identified hub genes and prognosis, respectively. The Gene Ontology (GO) and Kyoto Encyclopedia of Genes and Genomes (KEGG) detected the top different biological events and signaling pathways in elevated and depressed DEGs. The LASSO Cox regression model screened the highest predictive value markers of HCC prognosis. The receiver operating characteristic (ROC) curve and immunoinfiltration analysis were employed to analyze the role of these hub genes for HCC. Based on these methods, we identified several genes that could function as molecular markers to track the onset and progression of HCC.

## 2. Materials and Methods

### 2.1. Microarray Data

The GEO database, specifically, the GSE17548 and GSE25097 series based on GPL570 and GPL10687 platforms, respectively, was identified to screen for genes associated with liver cirrhosis and HCC [7]. The MINiML files, which contained raw data, including those for all of the platforms, samples, and GSE records, were obtained, and the extracted data were log transformed for standardization. Using preprocessCore, we normalized the data using the median method. The annotated information included in the platform was used to convert probes to gene symbols, which were then used in the normalization process. Probes that matched more than one gene were excluded from these datasets. Several probes were utilized to detect the expression value of each gene, and an average value from these was obtained. To eliminate the confounding effects of different batches, we used the “removeBatchEffect” function of the “limma” package in R. Boxplots were used to analyze the cleaned datasets. A PCA plot was constructed to demonstrate the differences in the datasets before and after the removal of the batch effects [8, 9].

### 2.2. Identification of DEGs

DEGs between HCC tissues and liver cirrhotic tissues were identified through GEO2R program. GEO2R, as a tool for interactive network, provides users with the ability to compare two or more datasets that are part of the GEO series to find DEGs [10]. The thresholds for statistical significance were set to log|fold change| > 1 and an adjusted *p* value of < 0.05.

### 2.3. Enrichment Analysis of DEGs

GO and KEGG databases were utilized as references, and the “clusterProfiler” R package carried out an analysis of enrichment [11]. To correct for multiple comparisons, the Benjamini–Hochberg approach was utilized, with a false discovery rate (FDR) < 0.05 indicating statistical significance.

### 2.4. Screening of Hub Genes

The STRING database (https://www.string-db.org/) was utilized to get a PPI network, with a score of 0.4 or higher for minimum participation in interactions [12]. The “Hubba” plug-in included in the Cytoscape program was used to identify and choose the top 10 hub nodes listed by degree [13].

### 2.5. Survival Analysis

The raw RNA-sequencing data and accompanying clinical information were obtained from the Cancer Genome Atlas (TCGA) database. Log-rank tests obtained *p* values, hazard ratios (HR), and 95% confidence intervals (CI) for the two groups (cirrhotic liver tissues and HCC tissues). These results were then used to plot Kaplan-Meier (KM) survival analysis to assess distinctions in survival between the cirrhotic liver and HCC tissue groups [14, 15].

### 2.6. Construction of Prognostic Signatures

To investigate the potential diagnostic utility of the hub genes identified, we carried out least absolute shrinkage and selection operator- (LASSO-) penalized Cox regression analysis [16]. The “glmnet” package in R package was used to develop a model for prognosis. A LASSO regression was carried out with the assistance of a cross-validation of 10 folds, with the penalty parameter (*λ*) adjusted to fulfil the optimal value. Findings of the LASSO regression were used as the basis for calculating risk ratings. Patients diagnosed with HCC who participated in the TCGA study were grouped into low-risk and high-risk categories based on the median risk score. A KM survival analysis was carried out to evaluate and contrast the variations in overall survival (OS) that were observed in the two groups [14, 17, 18].

### 2.7. Immunoinfiltration Analysis

The immunogene module of the TIMER tool (https://cistrome.shinyapps.io/timer/) was used to analyze correlations between the expression of hub genes and immunological infiltration (including infiltration levels of B cell, CD4 + T cell, CD8 + T cell, macrophage, neutrophil, and dendritic cell) in HCC tissues from the TCGA [19, 20].

## 3. Results

### 3.1. Screening for Differentially Expressed Genes

**Figure 1:**
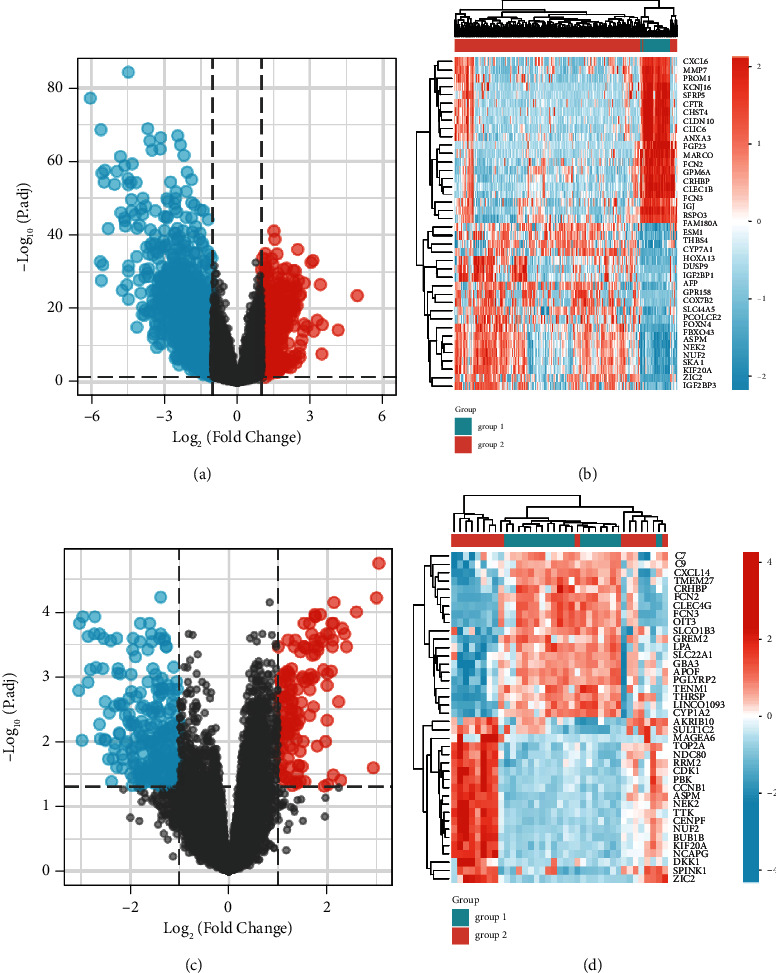
Differentially expressed genes (DEGs) between HCC and cirrhotic liver tissues identified in GSE25097 and GSE17549 datasets. (a) A total of 627 genes were found to be elevated and 1716 genes depressed in HCC tissues as compared to cirrhotic liver tissues in the GSE25097 dataset. (b) The expression profiles of each of the top 20 DEGs identified from the GSE25097 dataset. (c) A total of 149 genes were found to be elevated and 285 genes depressed in HCC tissues as compared to cirrhotic liver tissues in the GSE17548 dataset. (d) The expression profiles of each of the top 20 DEGs in the GSE17548 dataset.

Two datasets from the GEO database, namely, GSE25097 and GSE17548, were used to identify DEGs between cirrhotic liver and HCC tissues. We identified 2343 DEGs in GSE25097 dataset, of which 627 were elevated and 1716 were depressed in HCC tissues when compared to cirrhotic liver tissues (Figure 1(a)). In the GSE17548 dataset, we identified 434 DEGs, of which 149 were upregulated and 285 were downregulated in HCC tissues when compared to cirrhotic liver tissues (Figure 1(c)). The heatmap shows the expression levels of each of the top 20 DEGs (Figures 1(b) and 1(d)).

### 3.2. Screening for Hub Genes

**Figure 2:**
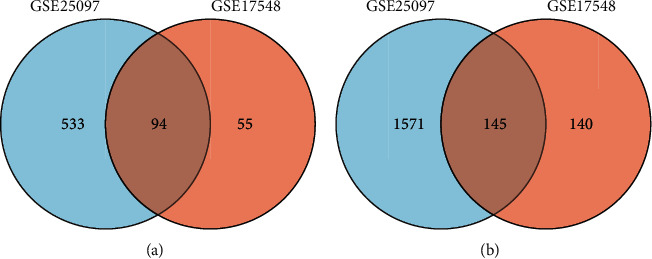
Identification of common genes from DEGs in the GSE25097 and GSE17548 datasets. (a) 94 genes were common upregulated, and (b) 145 genes were common downregulated.

**Figure 3:**
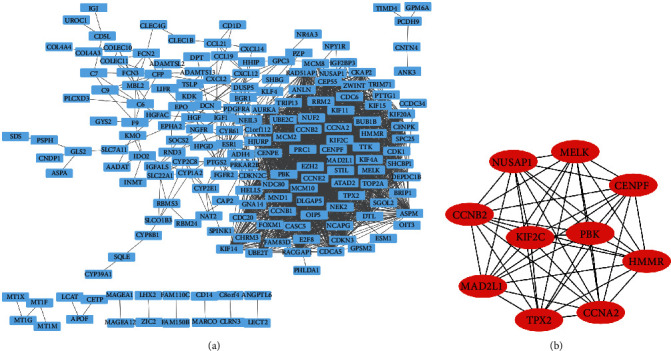
Screen for hub genes. (a) PPI network. (b) Top 10 hub genes were identified by CytoHubba.

To further analyze the common genes in the two datasets, Venn diagram was employed to find 94 common upregulated genes and 145 common downregulated genes in HCC tissues as compared to cirrhotic liver tissues (Figures 2(a) and 2(b)). Using STRING and Cytoscape, we analyzed these 239 DEGs to identify those with interaction scores > 0.4. PPI network obtained a total of 183 nodes and 2193 edges (Figure 3(a)). CytoHubba was utilized to get the top 10 hub genes, namely, BUB1B, MELK, MAD2L1, CCNB2, NUSAP1, RRM2, TTK, HMMR, CCNA2, and KIF2C (Figure 3(b)).

### 3.3. GO and KEGG Enrichment Analyses of DEGs

**Figure 4:**
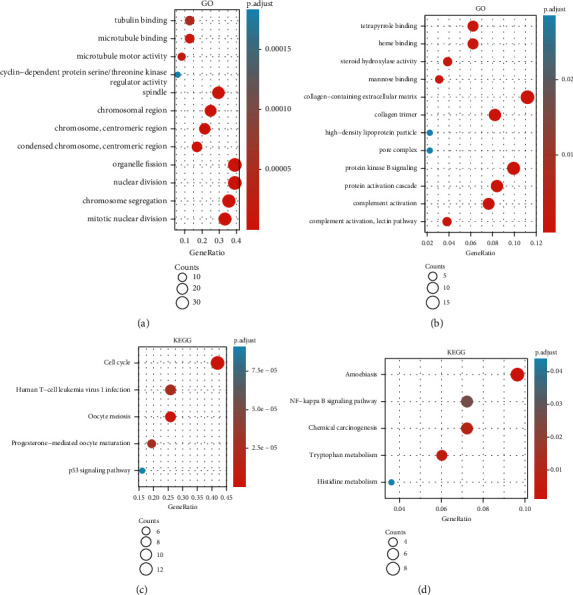
Functional enrichment analysis of hub genes in HCC. (a) GO analysis of DEGs in high expression samples. (b) GO analysis of DEGs in low expression samples. (c) KEGG analysis of DEGs in high expression samples. (d) KEGG analysis of DEGs in low expression samples.

To further examine the biological roles of the identified DEGs, we used the “clusterProfiler” package in R for GO and KEGG pathway enrichment analyses. The results of the GO analysis of upregulated DEGs indicated that this group contained genes related to biological processes (including mitotic nuclear division, chromosome segregation, nuclear division, and organelle fission), cellular components (including spindle, chromosomal region, chromosome, centromeric region, and condensed chromosome), and molecular functions (including tubulin binding, microtubule binding, microtubule motor activity, and cyclin-dependent protein serine/threonine kinase regulator activity) (Figure 4(a)). Analysis of the downregulated DEGs indicates that this group contains genes linked to biological processes (including complement activation, lectin pathway, complement activation, protein activation cascade, and protein kinase B signaling), cellular components (including pore complex, high-density lipoprotein particle, collagen trimer, and collagen-containing extracellular matrix), and molecular functions (including mannose binding, steroid hydroxylase activity, heme binding, and tetrapyrrole binding) (Figure 4(b)). In addition, KEGG analysis revealed that the elevated DEGs were intimately connected to the cell cycle, human T-cell leukemia virus 1 infection, oocyte meiosis, progesterone-mediated oocyte maturation, and the p53 signaling pathway (Figure 4(c)). The downregulated DEGs were connected to amoebiasis, NF-kappa B signaling pathway, chemical carcinogenesis, tryptophan metabolism, and histidine metabolism (Figure 4(d)).

### 3.4. Relationship between HCC Prognosis and Expression of Hub Genes

**Figure 5:**
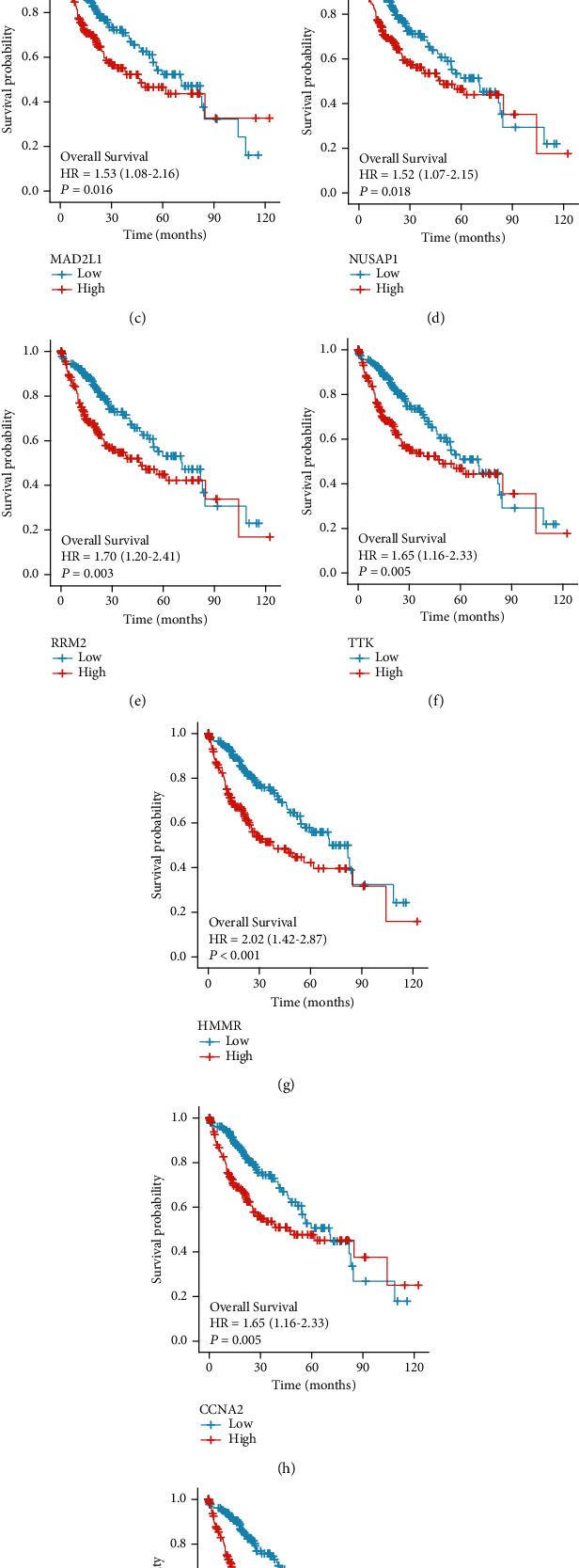
The prognostic potentials of the genes (a) BUB1B, (b) MELK, (c) MAD2L1, (d) NUSAP1, (e) RRM2, (f) TTK, (g) HMMR, (h) CCNA2, and (i) KIF2C were investigated. Patients diagnosed with HCC having higher levels of expression of these genes had lower overall survival statistics as compared to patients with lower levels of expression of these genes (logrank test, *p* < 0.05). Based on the Cox pH model, HR was determined, and the 95% CI was shown as a dotted line.

**Figure 6:**
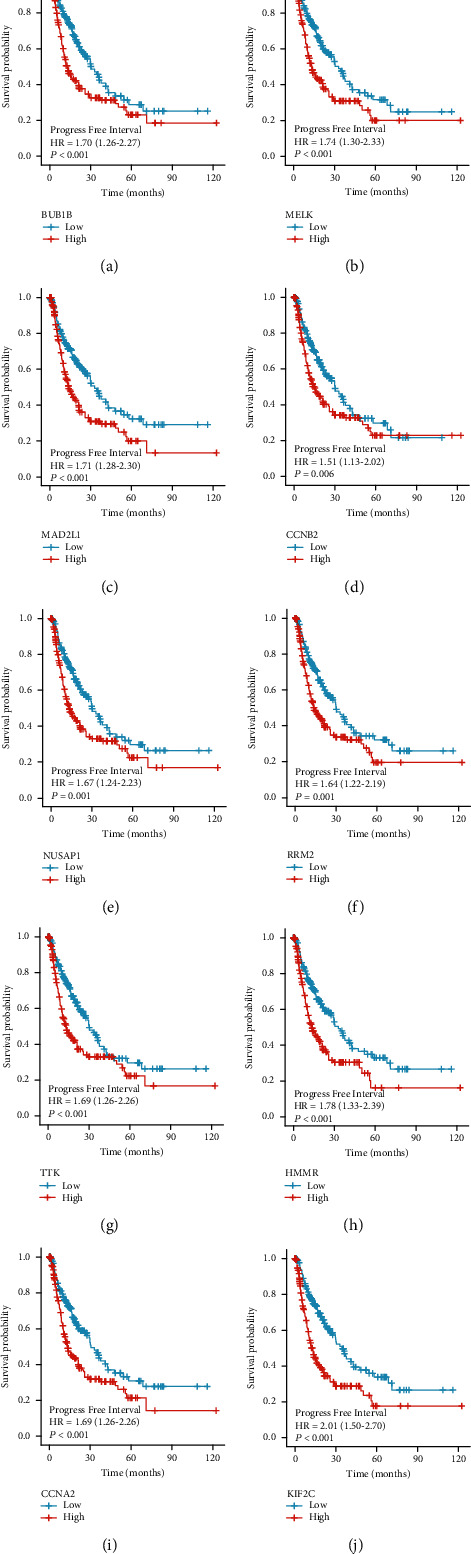
The prognostic potentials of the genes (a) BUB1B, (b) MELK, (c) MAD2L1, (d) CCNB2, (e) NUSAP1, (f) RRM2, (g) TTK, (h) HMMR, (i) CCNA2, and (j) KIF2C were investigated. Patients diagnosed with HCC having higher levels of expression of these genes had lower cancer-free intervals as compared to patients with lower expression levels of these genes (logrank test, *p* < 0.05). Based on the Cox pH model, HR was determined, and the 95% CI was shown as a dotted line.

**Figure 7:**
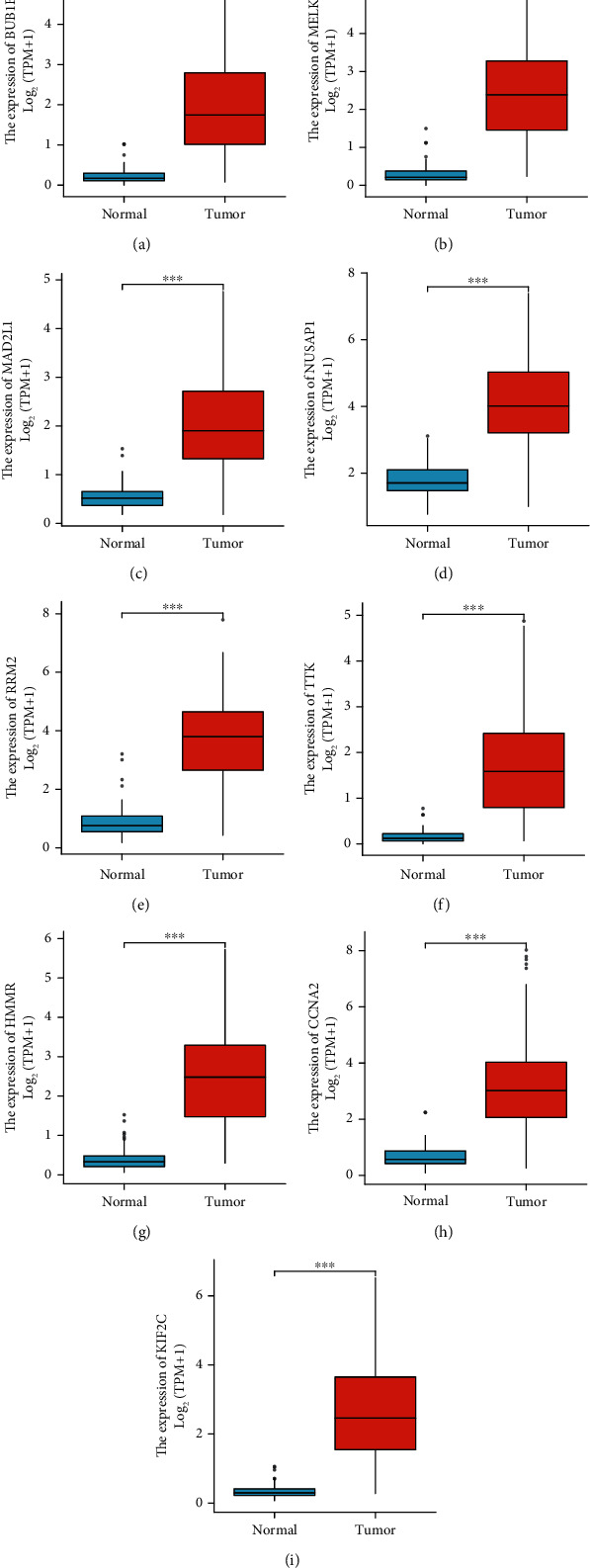
Differential expression analysis of hub genes in TCGA dataset consisting of HCC tissue samples and normal tissue samples. The expression levels of (a) BUB1B, (b) MELK, (c) MAD2L1, (d) NUSAP1, (e) RRM2, (f) TTK, (g) HMMR, (h) CCNA2, and (i) KIF2C in HCC tissues (*n* = 374) were significantly higher than those in normal tissues (*n* = 50).

**Figure 8:**
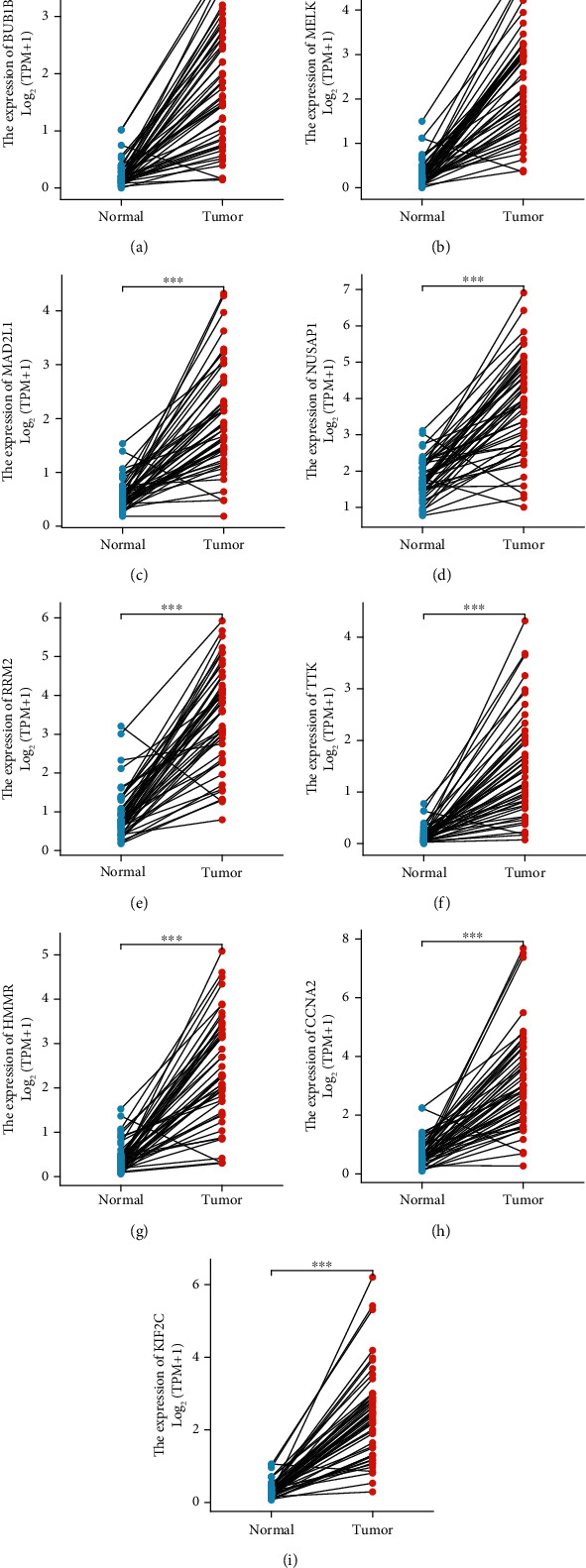
Differential expression analysis of hub genes in TCGA dataset consisting of HCC tissue samples and paired adjacent normal tissue samples. The expression levels of (a) BUB1B, (b) MELK, (c) MAD2L1, (d) NUSAP1, (e) RRM2, (f) TTK, (g) HMMR, (h) CCNA2, and (i) KIF2C in HCC tissues (*n* = 50) were significantly higher than those in paired adjacent tissues (*n* = 50).

A univariate Cox regression analysis was carried out to identify which hub genes were linked to HCC prognosis. We find that nine of the 10 identified hub genes (BUB1B, MELK, MAD2L1, NUSAP1, RRM2, TTK, HMMR, CCNA2, and KIF2C) showed prognostic significance (Figures 5 and 6). The expression profiles of these nine genes were then evaluated in 374 HCC tissue samples and 50 normal liver tissue samples obtained from the TCGA database. Our findings indicated that the expression levels of these nine hub genes in HCC tissues were significantly higher than those in normal tissues (Figures 7 and 8).

### 3.5. Construction of Prognostic Signatures of Hub Genes in HCC

**Figure 9:**
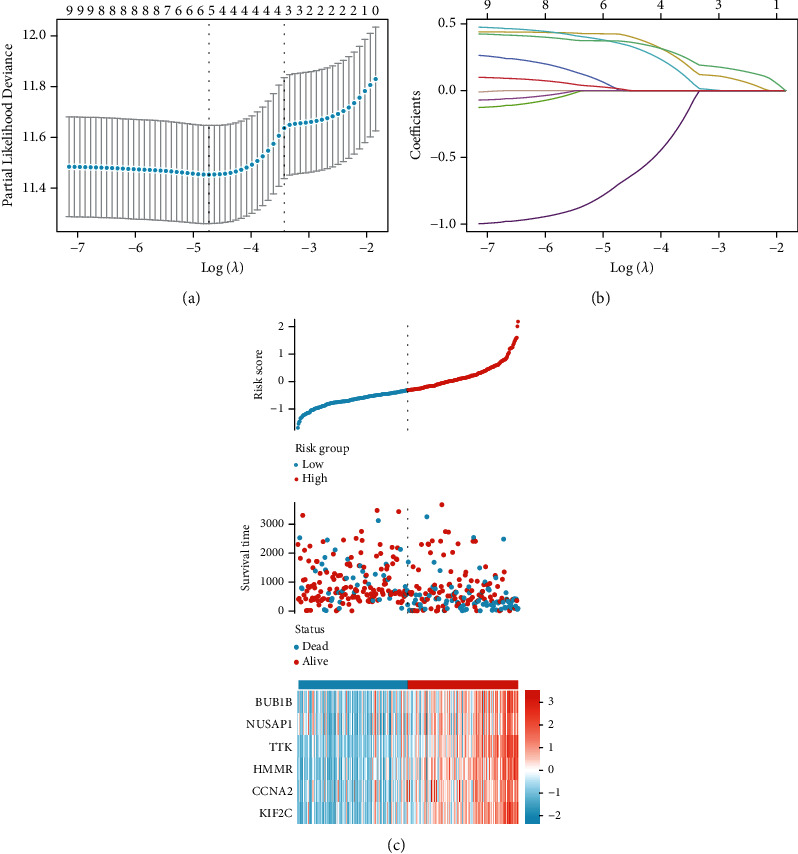
Clinical significance of these hub genes in HCC patients' data from TCGA. (a) Using the LASSO Cox regression model, the partial likelihood deviance versus log (*λ*) has been plotted. (b) Using the lambda parameter, chosen feature coefficients are shown. (c) Distribution of risk score, prognostic hub gene expression, and survival status of HCC patients.

The LASSO Cox regression model was utilized to choose genes with the highest predictive value as potential markers of HCC prognosis. The value (*λ* = 0.0088) was detected because it was the lowest when compared to the median of the sum of the squared residuals (Figures 9(a) and 9(b)). Six possible predictors (BUB1B, NUSAP1, TTK, HMMR, CCNA2, and KIF2C) were shown to have high predictive value for HCC prognosis. Patients diagnosed with HCC were split into two categories according to their risk scores.  Figure 9(c) depicts the distributions of risk scores, survival statuses, and expression levels of these six genes in the patient population (Figure 9(c)).

**Figure 10:**
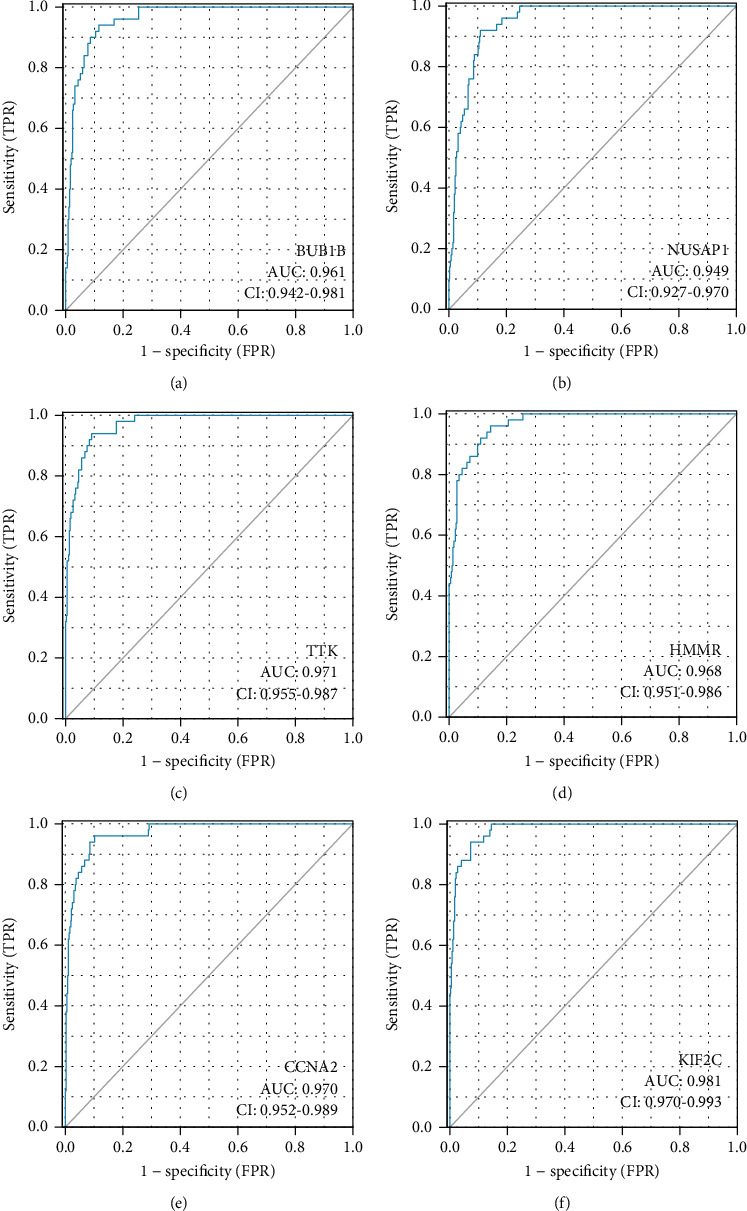
Receiver operating characteristic analysis (ROC) of (a) BUB1B, (b) NUSAP1, (c) TTK, (d) HMMR, (e) CCNA2, and (f) KIF2C in HCC patients' data (*n* = 424).

In TCGA, the data on 374 HCC samples with detailed clinicopathological information (Table 1) were evaluated for clinically relevant markers. These hub genes were measured at mRNA levels in HCC tissues and normal tissues, as well as the data was used to generate ROC curve. Our results indicated that BUB1B, NUSAP1, TTK, HMMR, CCNA2, and KIF2C were all upregulated in HCC at the mRNA levels. And the six hub genes had a high diagnostic value, with AUCs of 0.961, 0.949, 0.971, 0.968, 0.970, and 0.981, respectively (Figure 10).

### 3.6. Relationship between Hub Gene Expression and the Infiltration of Immune Cells

**Figure 11:**
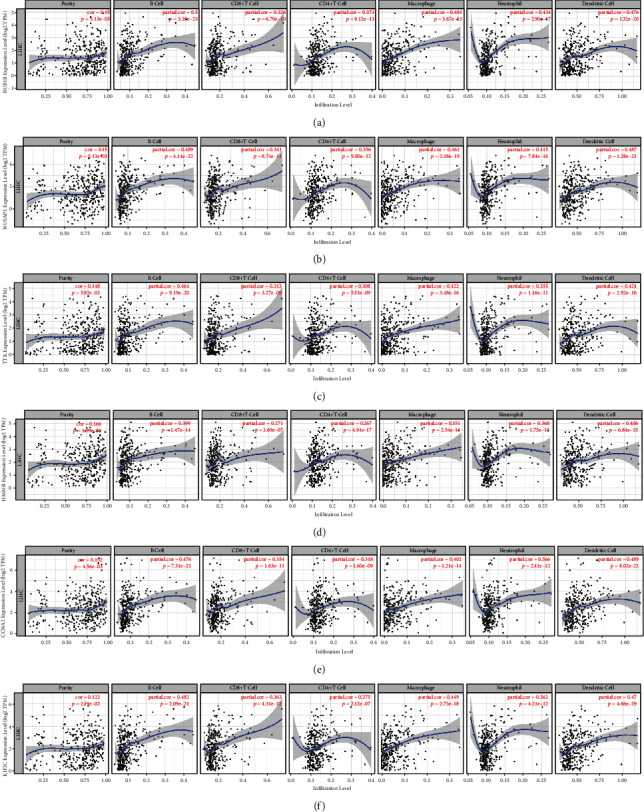
Relationships between immune infiltration in HCC tissues and expression levels of (a) BUB1B, (b) NUSAP1, (c) TTK, (d) HMMR, (e) CCNA2, and (f) KIF2C from the TIMER database.

It has been shown that tumor-associated fibroblasts in the stroma of the tumor microenvironment may affect a wide range of immune cells that infiltrate the tumor. The effects of the hub genes identified here on the recruitment of immune cells in the tumor microenvironment and hence on the prognosis of HCC are as yet unknown. To investigate this, we analyzed the connections between BUB1B, NUSAP1, TTK, HMMR, CCNA2, and KIF2C with immune infiltration in HCC and found that the expression levels of them were positively associated with the immune infiltration level of immune cells (Figure 11).

## 4. Discussion

Globally, HCC is the second deadliest and fifth most commonly occurring cancer [21]. The disease progression is quick with malignancy at a high level, which combined with low incidences of early detection, usually points to a bad prognosis. A high risk of developing HCC is associated with HBV or HCV infections, cirrhosis, and alcohol intake. Of these, cirrhosis is the most significant risk factor, since 80–90% of HCC patients usually suffered from cirrhosis [22, 23].

Patients diagnosed with HCC who undergo curative therapy in the early stages of the disease have significantly higher five-year survival rates [24]. However, the mechanism for liver cirrhosis progresses into HCC is as yet unknown, though there are two theories about this process. One theory assumes that liver cirrhosis itself is a precancerous stage that leads to HCC due to internal hepatic interstitial changes and modulations in cell proliferation. The second theory postulates that cirrhosis affects hepatocyte proliferation by making the cells more sensitive to carcinogenic factors in the external environment, which predisposes them to damage that leads to the development of HCC [25]. Since the rapid rate of cellular reproduction does not allow these cells sufficient time for DNA repair, mutations accumulate in newly produced cells, which pave the way to malignant transformation.

We tried to address this gap in knowledge by analyzing the differences in gene expression profiles between normal liver tissues, cirrhotic liver tissues, and HCC tissues. We screened several databases to get hub genes that may be responsible for the progression of cirrhosis into HCC. We found that genes linked to mitotic nuclear division, chromosomal segregation, nuclear division, and organelle fission are all intimately connected to this process. Through a series of bioinformatics analyses using data from two gene chip datasets (from cirrhotic liver and HCC tissues), we identified 239 DEGs, of which 94 were elevated and 145 were depressed. Using Cytoscape, we were able to identify ten possible hub genes from these DEGs. The genes with the highest prognostic potential were identified using the LASSO Cox regression model. The hub genes that we have identified are intricately connected to the incidence, progression, and prognosis of HCC and therefore may be very useful in the early detection and treatment of HCC.

We have identified BUB1B, NUSAP1, TTK, HMMR, CCNA2, and KIF2C as potential predictive markers for HCC. Previous studies indicate that expression levels of BUB1B, which is a spindle-assembly checkpoint gene [26], were highly upregulated in multiple myeloma patients and that these levels were strongly correlated with unfavorable outcomes [27]. Another marker, NUSAP1, which is a microtubule-associated protein involved in mitosis, is also known to participate in cell proliferation, apoptosis, and repairing DNA damage in glioblastoma multiforme cells [28]. The protein kinase encoded by the TTK gene is necessary for mitotic checkpoints as well as the DNA damage response [29]. Elevated HMMR in mouse mammary epithelium enhances the rate of Brca1-mutant carcinogenesis as it is involved in modifying the phenotype of tumor cell and tumor microenvironment [30]. The CCNA2 gene also plays an important role in HCC, as the HBV genome integrates into one of the CCNA2 introns and forms an in-frame chimeric fusion with CCNA2 [31]. The KIF2C gene, belonging to the Kinesin family, has been shown to be significantly overexpressed in several human malignancies [32].

Since mRNA is an essential component of all cells, including tumor cells, changes in mRNA levels of hub genes can be used as molecular indicators for a variety of disorders, including cancer [13, 33, 34]. We find that the AUCs for BUB1B, NUSAP1, TTK, HMMR, CCNA2, and KIF2C were all >0.9, which indicates the expression levels of these genes can be used to differentiate between HCC tissues and normal liver tissues. Besides, immune cells that have invaded a tumor are called tumor-infiltrating cells. These cells are a key part of the microenvironment of a tumor and are strongly related with carcinogenesis, progression, or metastasis. In our results, we found the six hub genes were all positively associated with the immune infiltration level of immune cells. All these results collectively suggested that these hub genes could serve as diagnostic molecular markers for HCC.

Considering that the predictive signature was developed and verified by the use of data from public databases, more experimental proof on top of the statistical evidence that we supplied will be required.

It is concluded that BUB1B, NUSAP1, TTK, HMMR, CCNA2, and KIF2C can be considered as potential novel molecular indicators for the onset and development of HCC, since they are linked to the transition from cirrhosis to HCC. This study will prove important reference for translational medicine scientists, liver disease specialists, and bioinformatics specialists.

## Figures and Tables

**Table 1:** Baseline clinical information.

Characteristic	Levels	Overall
*n*		374
T stage, *n* (%)	T1	183 (49.3%)
	T2	95 (25.6%)
	T3	80 (21.6%)
	T4	13 (3.5%)
N stage, *n* (%)	N0	254 (98.4%)
	N1	4 (1.6%)
M stage, *n* (%)	M0	268 (98.5%)
	M1	4 (1.5%)
Gender, *n* (%)	Female	121 (32.4%)
	Male	253 (67.6%)
Age, *n* (%)	≤60	177 (47.5%)
	>60	196 (52.5%)
AFP (ng/ml), *n* (%)	≤400	215 (76.8%)
	>400	65 (23.2%)
Vascular invasion, *n* (%)	No	208 (65.4%)
	Yes	110 (34.6%)
OS event, *n* (%)	Alive	244 (65.2%)
	Dead	130 (34.8%)
Child-Pugh grade, *n* (%)	A	219 (90.9%)
	B	21 (8.7%)
	C	1 (0.4%)
Age, median (IQR)		61 (52, 69)

## Data Availability

The datasets analyzed during the current study are available in TCGA (https://portal.gdc.cancer.gov/) and GEO repository (https://www.ncbi.nlm.nih.gov/geo/).
